# Conned by Conn’s: The Manifestation of Conn’s Syndrome Post-renal Transplant in a Patient with Polycystic Kidney Disease

**DOI:** 10.7759/cureus.7512

**Published:** 2020-04-02

**Authors:** Chaitanya Rojulpote, Ashwin Mathew, Manoj K Yarlagadda, Abhijit Bhattaru, Kiranmayi Vuthaluru

**Affiliations:** 1 Internal Medicine, The Wright Center for Graduate Medical Education, Scranton, USA; 2 Nuclear Cardiology & Cardiovascular Molecular Imaging, University of Pennsylvania, Philadelphia, USA; 3 Internal Medicine, University of Edinburgh, Edinburgh, GBR; 4 Internal Medicine: Gastroenterology, Mayo Clinic, Rochester, USA; 5 Radiology, Hospital of the University of Pennsylvania, Philadelphia, USA

**Keywords:** pckd, conns syndrome, renal transplant

## Abstract

We present the case of a 66-year-old African-American male with end-stage renal disease (ESRD) secondary to polycystic kidney disease (PCKD), with well-controlled hypertension. He was placed on peritoneal dialysis for two years before successfully undergoing a cadaveric renal transplant. There was an immediate graft function with no relevant postoperative complications. On regular follow-ups two months later, the patient now presents with worsening control of hypertension despite an increase in anti-hypertensive medications. Common causes of new-onset hypertension, such as renal artery stenosis, anti-calcineurin therapy, and allograft injury, were excluded. The patient’s biochemistry revealed the presence of hypokalemia, which was absent in previous reports. In light of this, plasma aldosterone and renin levels were sent and were found to be elevated: aldosterone: 50.4 ng/dL, renin: 0.4 ng/dL, aldosterone-renin Ratio (ARR): 126. In retrospect, a routine CT (computed tomography) scan performed in 2017 revealed an adrenal adenoma of 17 x 13 mm, which was diagnosed as an incidental finding at that time. A repeat CT scan was performed and showed no change in the size of the adenoma. In view of the new symptoms, the patient was started on spironolactone with little to no improvement in blood pressure and potassium levels. We present a case of Conn's syndrome in a patient with PCKD manifesting only after a renal transplant.

## Introduction

Primary hyperaldosteronism, or Conn's syndrome, is a relatively common condition encountered in clinical practice. It presents with typical signs of hypokalemia and hypertension, with the former occurring due to potassium wasting by the kidney and the latter occurring in conjunction with low plasma renin levels. While there are various etiologies of primary hyperaldosteronism, one of the most common causes is an adrenal adenoma. This case highlights the manifestation of Conn's syndrome in a patient with polycystic kidney disease (PCKD) after having undergone successful renal transplantation.

## Case presentation

We present the case of a 66-year-old African-American male with a history of end-stage renal disease (ESRD) secondary to polycystic kidney disease (PCKD) diagnosed at the age of 38 years. He was further diagnosed with hypertension at the age of 40, which was controlled with lisinopril 40 mg daily. He was initiated on peritoneal dialysis and had no complications while receiving the therapy. He subsequently received a cadaveric renal transplant, which had immediate graft function. He was induced on immunosuppression with basiliximab 20 mg intravenous (IV) followed with belatacept 10 mg/kg IV. His postoperative medications included mycophenolate mofetil 1000 mg every 12 hours, prednisone 5 mg daily, tacrolimus 5 mg daily with belatacept 5 mg/kg on a monthly basis. He was discharged on postoperative day four with no complications. His subsequent visits to the renal transplant clinic revealed worsening hypertension and symptoms of fatigue, cramps and generalized weakness in the second month postop, despite an increase in antihypertensive medications to nifedipine 90 mg every eight hours and metoprolol 25 mg every 12 hours. He was also found to have persistent hypokalemia despite aggressive potassium supplementation with 100 mEq. With the exception of persistent hypokalemia, all other lab values post-transplant were normal. He was on atorvastatin 10 mg, metoprolol 25 mg every 12 hours, mycophenolate mofetil 1000 mg every 12 hours, nifedipine 90 mg every eight hours, potassium chloride 100 mEq IV every 12 hours, prednisone 5 mg every day, and tacrolimus 2 mg every 12 hours at that time.

Given his long-standing history of hypertension and persistent hypokalemia, an aldosterone-renin ratio (ARR) test was ordered. His plasma aldosterone levels were elevated to 50.4 ng/dL and plasma renin activity was somewhat suppressed at 0.4 ng/dL under conditions of saline loading. His ARR was measured to be very high, i.e., 126. Upon further questioning, the patient revealed that a routinely ordered computed tomography (CT) scan from 2017 revealed an adrenal mass, which was said to be an incidental finding and periodic follow-ups were scheduled to assess progression. The patient was started on spironolactone 25 mg every 12 hours with mild improvement in his serum potassium levels. His other antihypertensives were gradually decreased to assess his response. His potassium has ranged from 3.0 to 3.8 mEq/L on 100 mEq of potassium chloride (KCl)/day and a small dose (25 mg every 12 hours) of spironolactone.

## Discussion

We present this case of a 66-year-old African American male with ESRD and PCKD who subsequently developed hypertension and persistent hypokalemia two months post-renal transplantation. A search for the common causes of new-onset hypertension was done and excluded which generally consists of renal artery stenosis of a transplanted kidney, iatrogenic anticalcineurin therapy, and chronic allograft injury [1].

**Figure 1:**
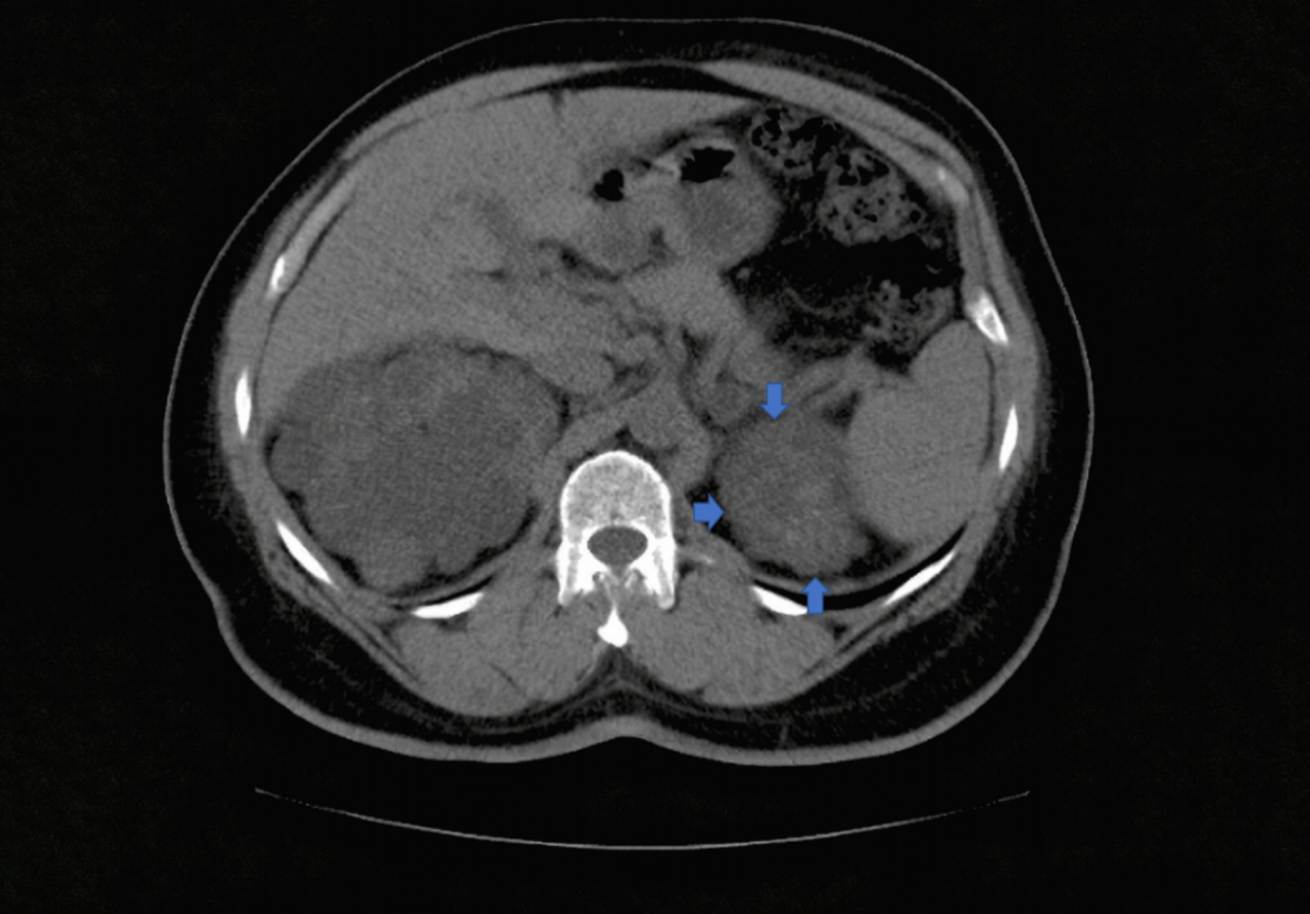
CT abdomen on transaxial section Blue arrows are marked to reveal the adrenal adenoma on the left side. CT: computed tomography

In view of the diagnostic uncertainty, an ARR was performed, the results of which were in keeping with a diagnosis of primary hyperaldosteronism, also more commonly known by the eponym, Conn’s syndrome. In hindsight, a routinely ordered CT scan in 2017 revealed an adrenal mass (Figure 1), which at that time was diagnosed as an incidentaloma in view of his electrolytes being within the range attributable to ESRD (normal to high potassium levels).

While the prevalence of primary hyperaldosteronism as a cause of secondary hypertension is more frequent (4%-5.9%), the incidence of primary hyperaldosteronism presenting after renal transplantation is exceedingly rare [2-3]. There are very few reports on such cases in published literature and of these, only two cases occurred in patients with PCKD [1,4-9]. We believe that this case highlights an important yet underrecognized cause of hypertension in post-renal transplant patients.

There are a couple of mechanisms underlying the so-called unmasking of Conn’s syndrome in post-renal transplant patients. It is postulated that the impaired potassium secretion by the kidneys in ESRD has masked the effects of hyperaldosteronism and only manifests itself (with hypokalemia) after renal transplantation once the kidney function is normalized [9]. Adding to the diagnostic conundrum, the difficulties of diagnosing primary hyperaldosteronism in patients with PCKD is two-fold. First, the presence of a large renal cyst may conceal a small adrenal adenoma [1]. Second, in the early stages of PCKD, there is increased renin release by the renal cysts, which thus contributes to secondary hyperaldosteronism [1,10].

Nevertheless, it is imperative to pick up Conn’s syndrome in patients with ESRD before renal transplantation is performed, as it represents a potentially reversible cause of hypertension and to prevent long-term cardiovascular complications [11]. Interestingly, it is also seen that high aldosterone levels in these patients have negative cardiovascular implications even in the context of normal blood pressure, further necessitating the need for early diagnosis [11]. A high index of suspicion is, therefore, required in these cases and a tendency towards relying on the mere presence of hypokalemia should be avoided. However, the recurrence of hypokalemia post-transplant should hint towards a diagnosis of primary hyperaldosteronism [1].

## Conclusions

In our patient, we believe that it may have been prudent to subject him for further testing at the time of diagnosis of the adrenal adenoma, specifically the use of simple blood tests such as the ARR. It would have also been worthwhile considering other tests, such as adrenal vein sampling, if the results of the ARR were inconclusive. This could have helped us plan for further management, i.e., adrenalectomy or medical management with spironolactone/eplerenone and would also provide a platform for ensuring closer follow-up post-renal transplantation to spot the diagnosis of Conn’s syndrome at its inception.
